# Neuroinvasion of *Toxocara canis*- and *T*. *cati*-larvae mediates dynamic changes in brain cytokine and chemokine profile

**DOI:** 10.1186/s12974-019-1537-x

**Published:** 2019-07-17

**Authors:** Patrick Waindok, Christina Strube

**Affiliations:** Institute for Parasitology, Centre for Infection Medicine, University of Veterinary Medicine Hannover, Buenteweg 17, 30559 Hanover, Germany

**Keywords:** Toxocarosis, Toxocariasis, Neuroinfection, Parasite, Roundworms, Neglected tropical disease, Multiplex bead array assay

## Abstract

**Background:**

Neurotoxocarosis (NT) is induced by larvae of the dog or cat roundworm (*Toxocara canis* or *T*. *cati*) migrating and persisting in the central nervous system of paratenic hosts, including humans, and may be accompanied by severe neurological symptoms. Host- or parasite-induced immunoregulatory processes contribute to the pathogenesis, but detailed data on pathogenic mechanisms and involvement of signalling molecules during cerebral *Toxocara* species infections are scarce.

**Methods:**

To elucidate alterations in immunomodulatory mediator pattern, comprehensive multiplex bead array assays profiling comprising 23 different cytokines and chemokines were performed during the course of *T*. *canis*- and *T*. *cati*-induced NT. To this end, cerebra and cerebella of experimentally infected C57Bl/6 J mice serving as paratenic host models were analysed at six different time points (days 7, 14, 28, 42, 70 and 98) *post infectionem* (pi).

**Results:**

Brain-body mass ratios of *T*. *canis* and *T*. *cati*-infected mice were significantly lower than those of the uninfected control group at day 14 pi, and also at day 28 pi for *T*. *canis*-infected mice. Both infection groups showed a continuous decrease of pro-inflammatory cytokine concentrations, including TNF-α, IFN-γ, GM-CSF and IL-6, in the cerebrum over the course of infection. Additionally, *T*. *canis* but not *T*. *cati*-induced neurotoxocarosis was characterised by significantly elevated levels of anti-inflammatory IL-4 and IL-5 in the cerebrum in the acute and subacute phase of the disease. The higher neuroaffinity of *T*. *canis* led to a prominent increase of eotaxin and MIP-1α in both the cerebrum and cerebellum, while in *T*. *cati*-infected mice, these chemokines were significantly elevated only in the cerebellum.

**Conclusions:**

The direct comparison of *T*. *canis*- and *T*. *cati*-induced NT provides valuable insights into key regulatory mechanisms of *Toxocara* species in paratenic hosts. The cerebral cyto-/chemokine milieu is shifted to a predominantly anti-inflammatory immune response during NT, possibly enabling both survival of the parasite and the neuroinfected paratenic host. Alteration of eotaxin and MIP-1α concentrations are congruent with the higher neuroaffinity of *T*. *canis* and species-specific tropism of *T*. *canis* to the cerebrum and *T*. *cati* to the cerebellum.

**Electronic supplementary material:**

The online version of this article (10.1186/s12974-019-1537-x) contains supplementary material, which is available to authorized users.

## Background

The roundworm species *Toxocara canis* (Werner 1782) and *T*. *cati* (Schrank 1788) are frequently occurring intestinal parasites of canids and felids. Adult worms live in the small intestine of their respective hosts, which contaminate the environment by excreting thousands of eggs per day with their faeces [1–3]. A variety of other species, including humans, may act as paratenic hosts upon ingestion of infective *Toxocara* larvae (L3). In paratenic hosts, larvae remain as infective L3 without further development [4, 5]. Migration and accumulation of larvae in tissues of paratenic hosts can cause the clinical picture of toxocarosis, one of the most common zoonotic infections worldwide [6]. The so called neurotoxocarosis (NT) is induced by larvae migrating and persisting in the central nervous system (CNS) [7], potentially causing meningitis, encephalitis, myelitis, cerebral vasculitis or behavioural disorders [7–9]. Furthermore, neuroinvasion of L3 may result in symptoms like motor impairments, as well as in behavioural alterations, memory impairment and cognitive dysfunctions and is additionally controversially discussed as a cause of neuropsychological disturbances like dementia or depression [9, 10]. Even though seropositivity rates in humans against *Toxocara* species range from 2 to 44% in Europe and 63 to 93% in tropical regions [6, 11–13], cases of NT tend to be underestimated due to nonspecific clinical signs.

Most cases of human NT are assumed to be caused by *T*. *canis* larvae and the majority of studies concerning toxocarosis and NT have focused on *T*. *canis* as the causative agent. However, *T*. *cati*-larvae have recently been reported as causative agents of human toxocarosis [14–16], and they show similar visceral larval migration patterns to *T*. *canis* larvae, even though *T*. *canis* exhibits a stronger neuroaffinity with a preference for the cerebrum, while *T*. *cati*-larvae prefer the cerebellum [17, 18]. Nevertheless, *T*. *cati* should not be neglected as a causative pathogen for (neuro-)toxocarosis in humans.

Several studies have addressed pathohistological alterations during NT, and infection with both *Toxocara* species results in, e.g. focal malacia, demyelination and spheroid formation [17–21]. Furthermore, larvae crossing the blood-brain barrier lead to injuries and increased permeability of this barrier during the course of infection [22]. However, the influence of *Toxocara* infections on inflammatory mediators as part of the cerebral immune response is scarcely described. Mediators like cytokines and chemokines play an important role in the coordination of the immune response by regulating inflammatory processes and modulating cellular activities [23]. The constitutive expression of cytokine-related genes in homeostatic cerebral tissues leads to the suggestion that these mediators participate in neuromodulation, development of the brain as well as the synaptic transmission [24]. In response to brain injury or infection of cerebral tissues, resident cells such as microglia, neurons, astrocytes and oligodendrocytes release various cytokines and chemokines, exerting pleiotropic immunomodulatory effects. An increased production of pro-inflammatory cytokines, like interleukin-1 (IL-1), IL-6, tumour necrosis factor-alpha (TNF-α) and interferon-gamma (IFN-γ), is beneficial, promoting clearance of invading pathogens and phagocytosis of cell debris, but prolonged inflammatory processes can result in detrimental neuronal injuries and neurodegeneration [25, 26]. Anti-inflammatory cytokines including IL-10, IL-4 and IL-5 play a regulating role in the immune response, for example by repressing the production of TNF-α, IFN-γ and IL-6 and promoting the alternative activation of macrophages [26]. Consequently, a complex network of interactions between pro- and anti-inflammatory cytokines determines the outcome of neuroinflammation. Hitherto, only a few studies focused on transcriptional changes of few selected cytokine- and brain injury-associated genes during the course of *T*. *canis*-induced brain infection [20, 21, 27, 28], but a comprehensive quantification of a broader range of cyto-/chemokines during the course of NT is still lacking. Furthermore, there is no information on cytokine or chemokine profiles during the course of *T*. *cati*-induced neurotoxocarosis, which differs from *T*. *canis*-induced NT in the severity of clinical symptoms and histopathological alterations in the mouse model [10, 18, 20, 29]. Therefore, the objective of this study was to investigate alterations in the complex cytokine network in brains of *T*. *canis*- and *T*. *cati*-infected mice as a model for human NT, to provide insights into cerebral immune responses and molecular host defence mechanisms against invading *Toxocara* larvae, which improves our understanding of the complex mechanisms and pathological processes during the course of neurotoxocarosis.

## Material and methods

### Infection of mice as a model for human neurotoxocarosis

Animal experiments were performed in accordance with the German Animal Welfare act in addition to national and international guidelines for animal welfare. Experiments were permitted by the ethics commission of the Institutional Animal Care and Use Committee (IACUC) of the German Lower Saxony State Office for Consumer Protection and Food Safety (Niedersaechsisches Landesamt für Verbraucherschutz und Lebensmittelsicherheit) under reference numbers 33.9-42502-05-01A038 and 33.12-42502-04-15/1869.

Eggs of *Toxocara canis* and *T*. *cati* were purified by a combined sedimentation-flotation technique from faeces of experimentally infected dogs and cats, respectively, and allowed to embryonate by incubation in tap water for about 4 weeks at 25 °C. Afterwards, eggs were stored at 4 °C until experimental infection of mice. C57BL/6JRccHsd mice (Harlan Laboratories, Horst, Netherlands) served as a model for neurotoxocarosis [30]. A total of 90 female mice were purchased at approximately 4 weeks of age and housed in Makrolon cages in a 12/12 h dark/light cycle receiving standard rodent diet (Altromin, Lage, Germany) and water ad libitum. At the age of 6 weeks, mice were randomly allocated to control and infection groups of 30 animals each. Animals of the *T*. *canis*- and *T*. *cati*-group were orally infected with 2000 embryonated *T*. *canis* or *T*. *cati* eggs, respectively, in a total volume of 0.5 ml tap water, whereas the control group received the same volume of the vehicle (tap water) only.

For subsequent analyses, each five mice of the *T*. *canis*- and *T*. *cati*-infection group as well as the control group were weighed and subsequently sacrificed by cervical dislocation at each time point (day post infection [pi]) in the acute phase (day 7 pi), the subacute phase (days 14 and 28 pi) and the chronic phase (days 42, 70 and 98 pi) of NT. Brains were carefully removed, divided into cerebrum and cerebellum, immediately snap frozen in liquid nitrogen and stored individually at − 150 °C until further analysis.

### Multiplex quantification of cytokines and chemokines

Individual cerebra and cerebella were weighed and ground to a homogeneous powder in liquid nitrogen using a mortar and pestle. Subsequently, 15 ± 2 mg of homogenised tissue was weighed into 1.5 ml tubes. Proteins were extracted using the Bio-Plex® Cell Lysis Kit (Bio-Rad, USA) according to the manufacturer’s instructions. Briefly, tissues were mixed with 500 μl lysing solution containing Bio-Plex® cell lysis buffer, Bio-Plex® factors 1 and 2 as well as phenyl-methylsulfonyl fluoride (PMSF, 500 mM; Sigma–Aldrich, USA) and grounded 20 times with small pellet pestles. Homogenates were frozen overnight at − 80 °C and thawed and centrifuged at 4500×*g* for 4 min at 4 °C the following day. The supernatant was collected and stored at *−* 80 °C until further analysis. The protein concentration was determined with the Bio-Rad DC Protein Assay (Bio Rad, USA). By using the supplied bovine gamma globulin as standard, sample absorbances were read at 750 nm using a spectrophotometer (PowerWave™ 340, BioTek, USA) and Gen5™ software (BioTek, USA).

The multiplex bead array assay (Bio-Plex Pro Mouse Cytokine 23-plex Assay [Bio Rad, USA]) was used for detection and quantification of cerebral and cerebellar concentrations of 23 different cyto-/chemokines, namely the cytokines granulocyte-colony stimulating factor (G-CSF), granulocyte-macrophage colony-stimulating factor (GM-CSF), IFN-γ, IL-1α, IL-1β, IL-2, IL-3, IL-4, IL-5, IL-6, IL-9, IL-10, IL-12(p40), IL-12(p70), IL-13, IL-17A and TNF-α, and the chemokines eotaxin (CCL11), keratinocyte chemoattractant (KC; CXCL1), monocyte chemoattractant protein 1 (MCP-1; CCL2), macrophage inflammatory protein 1-alpha (MIP-1α; CCL3), MIP-1β (CCL4) and regulated on activation, normal T cell expressed and secreted (RANTES; CCL5). The assay was conducted according to the manufacturer’s protocol with each sample analysed in duplicate. For washing steps, a Bio-Plex Handheld Magnetic Washer (Bio Rad, USA) was used. All samples were diluted in sample diluent and stabilised with 0.5% (*w*/*v*) bovine serum albumin to a final protein concentration of 900 μg/ml. A Luminex 200™ system (Luminex Corporation, The Netherlands) was used for detection and quantification of analytes. Data were processed with the Bio-Plex Manager software (version 6.1, Bio Rad, USA), with a five-parameter logistic regression model (5PL) for standard curve creation.

### Data analysis

Normality distribution of all sample sets were analysed by the Kolmogorov-Smirnov test. The One-way ANOVA with subsequent unpaired *t* test for normally distributed variables and the Kruskal-Wallis test with subsequent Mann-Whitney *U* test for skewed distributions were used to reveal significant differences between the three groups at each time point. If a metabolite could not be detected in one of the study groups, the lower limit of quantification was used for statistical analyses, which were conducted with the GraphPad Prism™ software (version 6.03, GraphPad Software, USA). In all analyses, *P* ≤ 0.05 was considered as statistically significant.

Ratios of cytokines and chemokines between the infection groups and uninfected control group for each time point were calculated by dividing each individual value of the *T*. *canis*-, *T*. *cati*- and uninfected control group by the mean value of the uninfected control group. The obtained fold-change values were log2 transformed and the means of the log2 transformed fold-changes were visualised as heatmap using MeV (version 4.9.0, TM4 Software suit [http://mev.tm4.org]) [31].

## Results

### Clinical assessment

Infected mice showed varying degrees of clinical symptoms and neurobehavioural alterations as described by Janecek et al. [10]. Mice infected with *T*. *canis* developed earlier and more severe symptoms than those infected with *T*. *cati*, comprising ataxia to paresis and paraplegia, incoordination, balance problems and reduced fear- and flight-related behaviour.

### Body and brain mass

Body and brain mass and the brain/body mass ratios of *Toxocara*-infected groups vs. the uninfected group and corresponding *P* values are provided as Additional file 1. The brain/body mass ratio of *T*. *canis*-infected mice was significantly decreased at days 14 pi (*P* = 0.0038) and 28 pi (*P* = 0.0353) in comparison to the uninfected control group, whereas *T*. *cati*-infected mice only showed a significant reduction at day 14 pi (*P* = 0.0024). The cerebra of uninfected control mice accounted for 1.2% to 1.8% of the total body mass. Cerebra of *T*. *canis*-infected animals accounted for 1.3% to 1.5% of the total body mass and showed a significant reduction of the cerebrum/body mass-ratio at days 14 and 28 pi (*P* = 0.0005 and *P* = 0.0396) and a significant increase at day 70 pi (*P* = 0.0079). The cerebra of *T*. *cati*-infected mice ranged from 1.3 to 1.6% of the total body mass, but a statistically significant decrease of the cerebrum/body mass-ratio was only detected at day 14 pi (*P* = 0.0003). The cerebellum/body mass-ratio of all three groups ranged between 0.4 and 0.7% and no statistically significant differences could be detected between the uninfected and infected groups.

### Analysis of cytokines and chemokines during NT

**Fig. 1:**
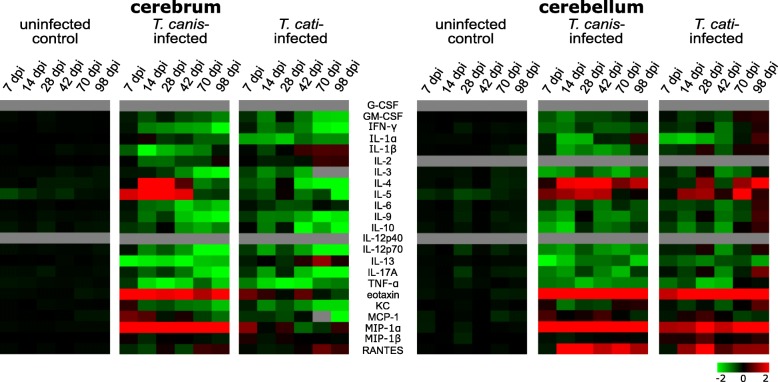
Heatmap displaying the mean fold-change of each cytokine and chemokine during the course of *T*. *canis*- and *T*. *cati*-induced neurotoxocarosis. Metabolites below the quantification limit are marked in grey

Cerebral and cerebellar levels of cytokines and chemokines were quantified by multiplex bead array assay throughout the course of *T*. *canis*- and *T*. *cati*-induced neurotoxocarosis. An overview of the alterations of cytokines and chemokines in terms of their fold-changes in infected compared to uninfected control mice is given in Fig. 1. Cytokine and chemokine concentrations as well as the *P* values regarding the comparison to uninfected control mice are displayed as Additional file 2 (cerebrum) and Additional file 3 (cerebellum).

### Course of cytokines during NT

**Fig. 2:**
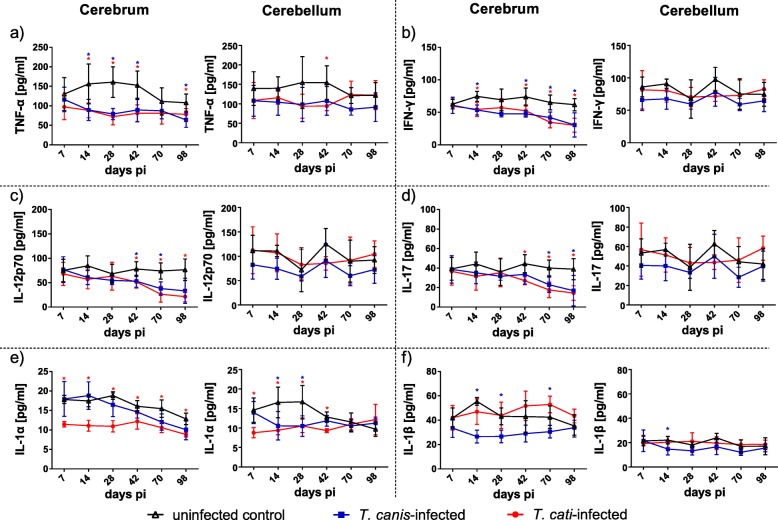
Concentrations of different pro-inflammatory cytokines in cerebra and cerebella of *T*. *canis*-, *T*. *cati*- and uninfected control mice. Red asterisks indicate statistically significant differences (*P* ≤ 0.05) between *T*. *cati*-infected and uninfected mice, blue asterisks between *T*. *canis*-infected and uninfected mice

From the 17 targeted cytokines, solely IL-12(p40) and G-CSF could not be detected in any of the three cohorts during the study period (day 7 to 98 pi). Variations of pro-inflammatory cytokines in the brain during the course of infection are illustrated in Fig. 2. Compared to uninfected mice, concentrations of pro-inflammatory TNF-α in the cerebra of *T*. *canis*- and *T*. *cati*-infected animals were significantly reduced over almost the entire study period (Fig. 2a), whereas in the cerebellum, a significant reduction was detected only for *T*. *cati*-infected mice at day 42 pi. During chronic NT, in both infection groups, a continuous decrease of IFN-γ, IL-12(p70) and IL-17 was observed in the cerebrum, but not in the cerebellum (Fig. 2b–d). Differences in the pro-inflammatory cytokine secretion between *T*. *canis*- and *T*. *cati*-infected cerebra were observed for IL-1α and IL-1β (Fig. 2e, f). Cerebral concentrations of IL-1α of *T*. *cati*-infected mice were significantly decreased during the whole study period, whereas those of *T*. *canis-*infected mice remained unaffected (Fig. 2e). Levels of IL-1α were also significantly decreased in the cerebella of *T*. *cati*-infected mice until the beginning of the chronic phase (day 42 pi), and in *T*. *canis*-infected mice in the subacute phase (days 14 and 28 pi). The concentration of IL-1β in the cerebra of *T*. *canis*-infected mice was reduced during the whole course of infection with statistically significant differences at days 14, 28 and 70 pi, while those of *T*. *cati*-infected mice did not show any significant IL-1β alterations (Fig. 2f). Similarly, cerebella of both infection groups remained unaffected (except day 14 pi in *T*. *canis*-infected mice).

**Fig. 3:**
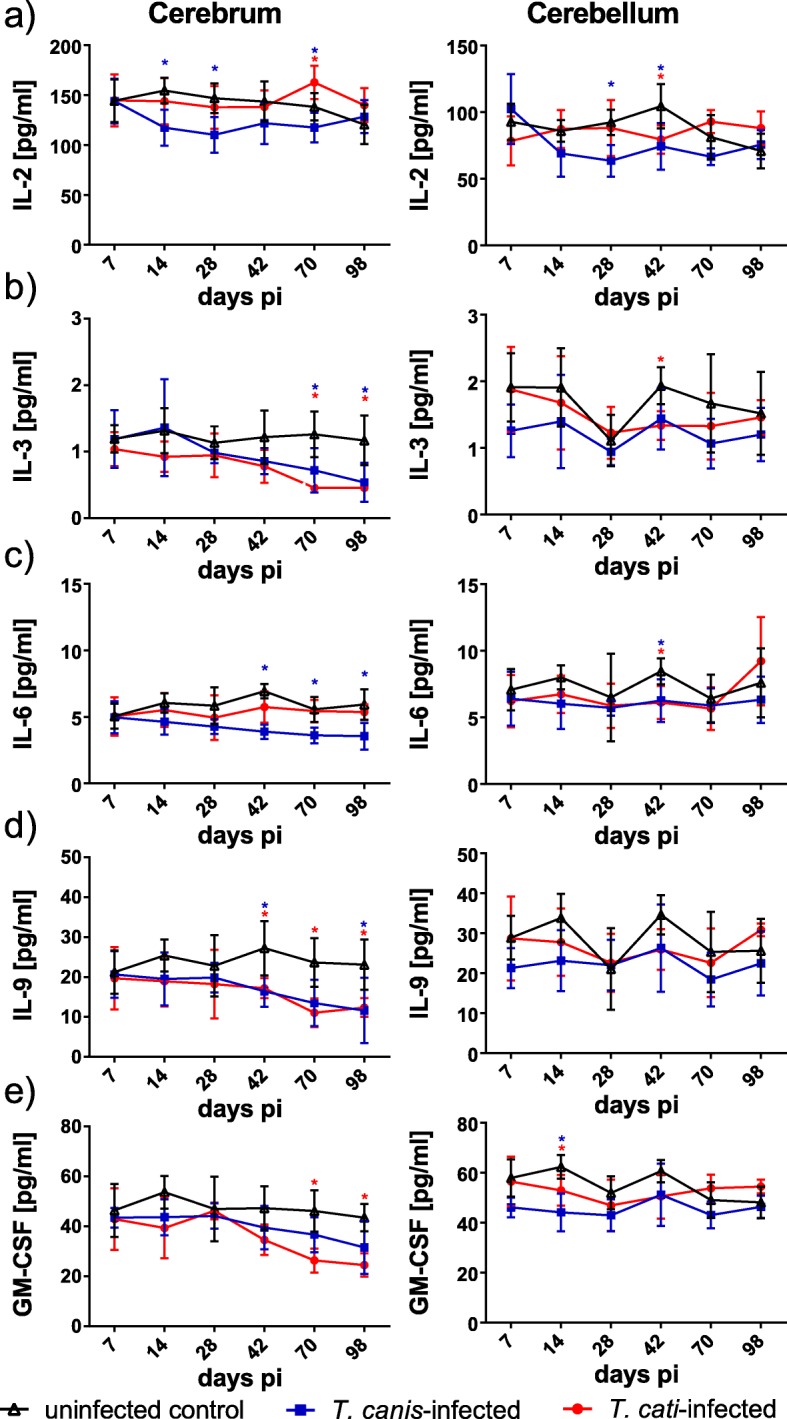
Concentrations of pro- or anti-inflammatory cytokines in cerebra and cerebella of *T*. *canis*-, *T*. *cati*- and uninfected control mice. Red asterisks indicate statistically significant differences (*P* ≤ 0.05) between *T*. *cati*-infected and uninfected mice, blue asterisks between *T*. *canis*-infected and uninfected mice. As IL-3 could not be detected in the cerebrum of *T*. *cati*-infected mice at days 70 and 98 pi, dotted lines represent the respective lower limit of quantification

Secretion of IL-2 was significantly decreased in cerebra of *T*. *canis*-infected mice at days 14, 28 and 70 pi, and in cerebella on days 28 and 42 pi. *T*. *cati*-infected mice showed altered IL-2 concentrations at one day only (Fig. 3a). By contrast, the other measured cytokines (IL-3, IL-6, IL-9 and GM-CSF) showed significantly decreased cerebral levels in the chronic phase of NT (as of day 42/70 pi). While IL-3 and IL-9 were decreased in both infection groups (Fig. 3b, d), IL-6 was only significantly decreased in *T*. *canis*-infected (Fig. 3c) and GM-CSF only in *T*. *cati*-infected mice (Fig. 3e). In cerebella, this pattern was not evident.

**Fig. 4:**
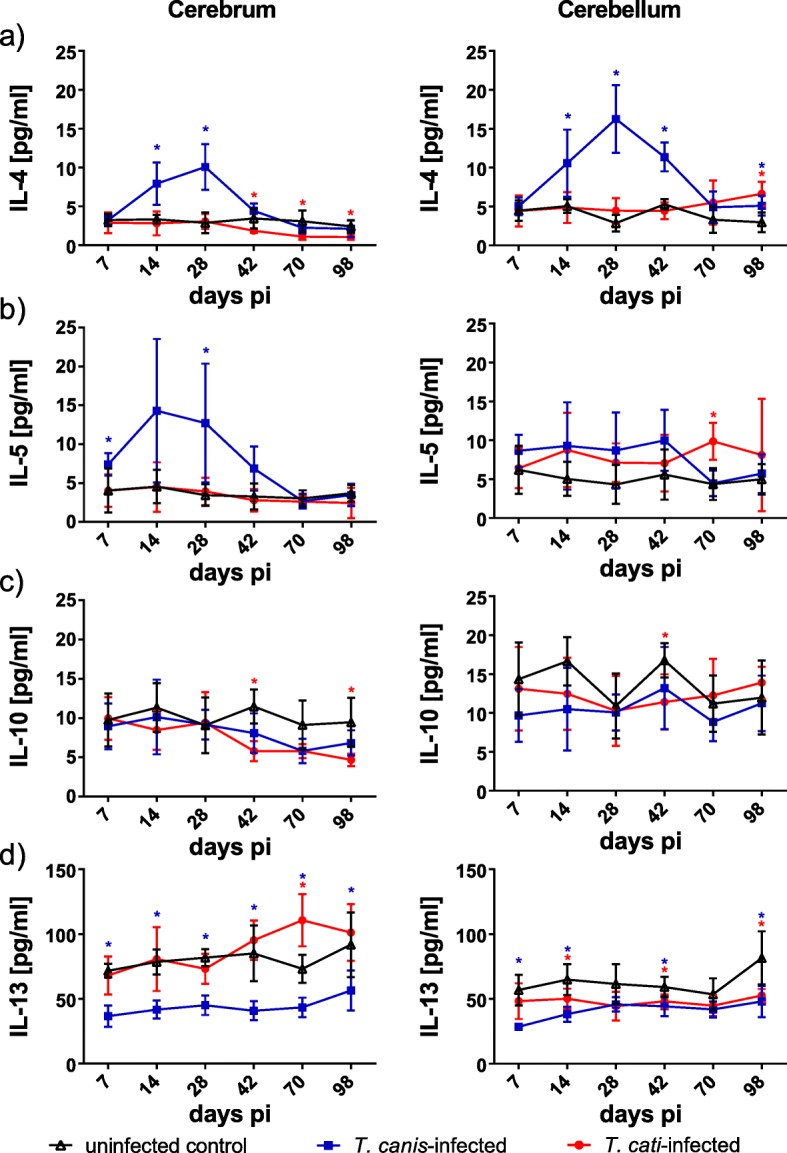
Concentrations of different anti-inflammatory cytokines in cerebra and cerebella of *T*. *canis*-, *T*. *cati*- and uninfected control mice. Red asterisks indicate statistically significant differences (*P* ≤ 0.05) between *T*. *cati*-infected and uninfected mice, blue asterisks between *T*. *canis*-infected and uninfected mice

Analysis of anti-inflammatory cytokines revealed a peak of IL-4 and IL-5 in cerebra of *T*. *canis*-infected mice in the subacute phase of infection, decreasing to homeostatic conditions as of day 42 pi (Fig. 4a, b). In the cerebella, IL-4 levels in *T*. *canis*-infected mice also showed a peak in the subacute phase, but lasting until day 42 pi (Fig. 4a), whereas IL-5 did not show a peak pattern. In *T*. *cati*-infected mice, none of the two cytokines showed a peak pattern, neither in the cerebra nor the cerebella. A decreased anti-inflammatory cerebral cytokine response was observed for IL-10 in the chronic phase with significantly reduced concentrations in the *T*. *cati*-infected mice at days 42 and 98 pi, whereas cerebellar concentrations remained largely unaffected (Fig. 4c). Concentrations of IL-13 were significantly decreased during the whole study period in both brain parts in *T*. *canis*-infected mice. In *T*. *cati*-infected mice, this pattern was only indicated in the cerebellum, whereas cerebral IL-13 concentrations were similar to those of the uninfected control in the acute and subacute phase but seemed to rise during chronic neurotoxocarosis with a significant increased value at day 70 pi (Fig. 4d).

### Course of chemokines during NT

**Fig. 5:**
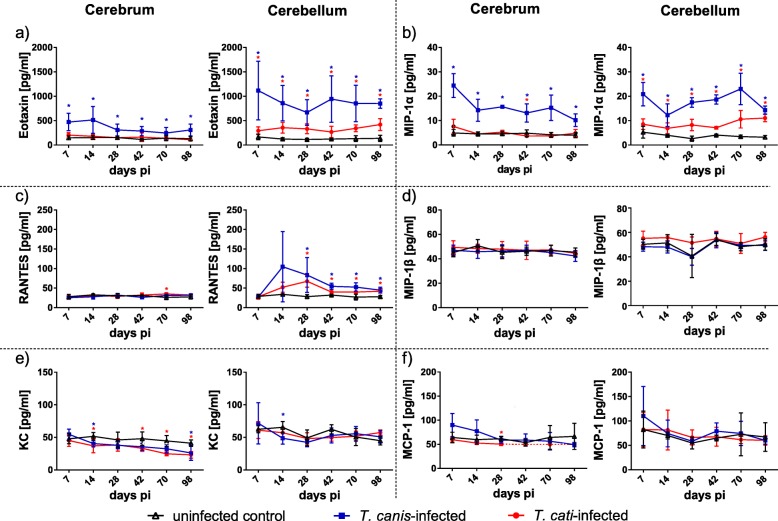
Concentrations of chemokines in cerebra and cerebella. Red asterisks indicate statistically significant differences (*P* ≤ 0.05) between *T*. *cati*-infected and uninfected mice, blue asterisks between *T*. *canis*-infected and uninfected mice. As MCP-1 could not be detected in the cerebrum of *T*. *cati*-infected mice at days 42, 70 and 98 pi, dotted lines represent the respective lower limit of quantification

The six targeted chemokines were detected in all three study groups. Significantly increased concentrations were obvious for eotaxin (CCL11; Fig. 5a) and MIP-1α (CCL3; Fig. 5b) in both the cerebrum and the cerebellum of *T*. *canis*-infected mice during the entire study period, whereas in *T*. *cati*-infected mice, this pattern was only observed in the cerebellum, and was less pronounced as compared to the *T*. *canis*-infection group. Increased cerebellar concentrations were also found for the chemokine RANTES, showing significantly elevated values in both infection groups as of the subacute phase of infection (day 28 pi), while cerebral concentrations were almost unaffected (Fig. 5c). By contrast, KC (CXCL1; Fig. 5e) presented significantly reduced cerebral values throughout almost the entire study period in *T*. *cati-*infected mice and selectively in *T*. *canis-*infected mice, while cerebellar concentrations did not follow this trend. Similarly, concentrations of MCP-1 (CCL2; Fig. 5f) and MIP-1β (CCL4; Fig. 5d) did not show apparent changes in both brain parts; however, MCP-1 levels were below the detection limit as of day 42 pi in cerebra of *T*. *cati*-infected mice.

## Discussion

The CNS manifestation of *Toxocara* species in paratenic hosts can be associated with severe pathology, for instance meningoencephalitis, myelitis and cerebral vasculitis [9]. Furthermore, neurodegenerative and neuropsychiatric disorders as well as behavioural alterations have been described as a consequence of NT [20, 28, 32]. The infection is accompanied by inflammatory processes which may have an effect on brain weight. In *Toxoplasma gondii*-infected C57BL/6 mice, Gatkowska et al. [33] described a transient increase in brain mass 3 weeks pi supposedly due to accumulation of inflammatory cells, followed by a decrease 6 weeks pi, which might be attributed to repression of brain inflammatory reactions [33]. As the brain mass is dependent on the body mass, the ratio between brain and body mass was used in the present study to obtain a more robust parameter. In contrast to *T*. *gondii* infection, *T*. *canis*- and *T*. *cati*-infected C57BL/6 mice showed significantly lower brain-body mass ratios than the uninfected control mice at day 14 pi, and in the case of *T*. *canis*-infection also at day 28 pi (day 21 pi was not examined). This decrease in the brain-body mass ratio was related to a decrease in the cerebrum-body mass ratio, while the cerebellum-body mass ratio remained unaffected. As of 6 weeks (day 42) pi, brain-body mass ratios of *Toxocara*-infected and uninfected mice remained the same.

The immune response of the CNS differs unequivocally to other tissues, with its own residential immune network, consisting mainly of microglia and astrocytes [34]. Neuroinflammation elicits, for example, the activation of microglia, which may eliminate invading pathogens. However, sustained, excessive or inappropriate inflammatory reactions in the brain can be detrimental, resulting in chronic neurodegenerative disease [26]. To prevent a damaging outcome, these mechanisms are usually balanced and regulated by anti-inflammatory and neuroprotective mediators.

The potent pro-inflammatory cytokines TNF-α, IFN-γ and IL-6 play a key role in initiating and sustaining neuroinflammation. TNF-α, for instance, induces apoptotic cell death in oligodendrocytes and the blockage of microglia-dependent TNF-α secretion suppresses inflammatory and demyelinating processes in experimental auto-immune encephalomyelitis [34, 35, 36]. These pro-inflammatory cytokines are also involved in the immune response against neuroinvasive parasites. Cerebral infections with the nematode *Angiostrongylus cantonensis* led to elevated brain levels of TNF-α, IFN-γ and IL-6 mRNA in mice as a model for paratenic hosts [37]. Regarding cerebral *Toxocara* infections, transcript levels of IFN-γ were elevated in *T*. *canis*-infected from day 3 to 42 pi in BALB/c and NIH mice [27] and transcript levels of IL-6 and TNF-α were increased at week 5 and 16 pi in male Swiss albino mice [21]. In contrast to elevated mRNA transcription in these previous studies, the present study showed a decrease of these pro-inflammatory metabolites as directly measured by multiplex bead array assay in the cerebral tissue of *T*. *canis*-infected C57BL/6 mice. This decrease became apparent as of the subacute phase (day 14 pi) for TNF-α and IFN-γ, and the chronic phase (day 42 pi) for IL-6. The deviant results between mRNA transcription data and the cytokine protein concentrations measured in the present study may indicate an active manipulation of the hosts’ immune response by *Toxocara* larvae, as hypothesised for *T*. *canis* excretory–secretory (TES) products, containing peptides, enzymes, lectins and mucins [38]. Indeed, macrophages derived from *T*. *canis*-infected mice showed a diminished production of TNF-α and IL-12 [39].

However, altered cytokine profiles as part of the brain immune response during NT have only been described for *T*. *canis*, but not for *T*. *cati* as causative agent. Both *Toxocara* species show a deviant species-specific tropism described by Janecek et al. [18], as larvae of *T*. *canis* exhibit a stronger affinity to the brain, particularly the cerebrum, while *T*. *cati* larvae prefer the cerebellum. Therefore, this study compared cyto- and chemokine concentration patterns during *T*. *canis*- and *T*. *cati*-induced NT. Regarding pro-inflammatory cytokines including TNF-α, IFN-γ, IL-12p70 and IL-17, similar patterns were observed in *T*. *canis*- and *T*. *cati*-infected mice in general. However, the cerebral content of IL-1α and IL-1β, which contribute to a wide range of neurodegenerative conditions as well as acute and chronic neuroinflammatory processes [40], differed between both infection groups. While IL-1β but not IL-1α was significantly reduced in the cerebra of *T*. *canis*-infected mice, IL-1α was predominantly reduced in *T*. *cati*-infected cerebra. Interestingly, IL-2 which is known to modulate expression of receptors for other cytokines and transcription factors and therefore can potentially prime and maintain Th1 and Th2 differentiation [41], showed a similar pattern to IL-1β. Pathogen-specific responses were also observed for IL-6 and GM-CSF, which were significantly reduced during chronic NT in cerebra of only *T*. *canis*-infected or *T*. *cati*-infected mice, respectively, while IL-3 and IL-9 were decreased in both infection groups.

Differences between *T*. *canis*- and *T*. *cati*-infected mice were also observed for anti-inflammatory type 2 cytokines. In general, helminth infections commonly induce a strong Th2 response, which is orchestrated by many different cell types and characterised, among others, by the secretion of IL-4, IL-5 and IL-13 [42, 43]. Even though IL-4 and IL-13 may have similar effects on immune cells [44], both cytokines are differentially regulated in mice during an infection with the rodent hookworm *Nippostrongylus brasiliensis* [45] as well as in the present study. Nevertheless, a *T*. *canis*-induced CD4^+^ Th2 activity, associated with the production of specific antibodies, has been described [46]. Interestingly, a prominent peak pattern of IL-4 and IL-5 was noted in cerebra of *T*. *canis*-infected mice in the acute and subacute phase of infection. Noteworthy, this pattern was absent in *T*. *cati*-infected mice, but IL-4 levels decreased from the beginning of the chronic phase (day 42 pi). In the cerebellum, *T*. *canis*-infected mice showed an IL-4 peak pattern comparable to the cerebra, while IL-5 was unaffected. Mice infected with *T*. *cati* revealed only selective increased levels of these Th2 cytokines and only in the chronic phase, though this pathogen prefers the cerebellum. These findings might reflect that the progression of *T*. *cati*-induced NT generally appears delayed compared to *T*. *canis*-infections, with a less severe pathogenesis in terms of clinical symptoms, histopathological alterations and deviant behavioural changes [10, 18, 20, 47]. It is conceivable that the TES product secreted by *T*. *cati* differs from that of its’ congener *T*. *canis*. Larvae of *T*. *cati* might mimic host antigenic components in order to escape or manipulate the hosts’ immune response and diminish neuroinflammation. Alternatively, the low type 2 response in *T*. *cati* infected brains might be attributed to generally lower larval numbers in the brain [17, 18].

The transition period between the subacute and the chronic phase of infection seems to be a pivotal period in NT. In the present study, the overall picture of pro-inflammatory and pleiotropic cytokine secretion during NT shows that only few cytokines are affected in the acute/subacute phase of infection, while almost all of these mediators decreased in the chronic phase, either in *T*. *canis*- or *T*. *cati*-infected mice or both. Additionally, *T*. *canis*-infection led to a prominent increase of anti-inflammatory IL-4 and IL-5 in the acute/subacute phase of infection which reached homeostatic levels with beginning of the chronic phase (day 42 pi). At this point in time, a plethora of differentially transcribed genes, mainly associated with “immune and defence response”, were detected in cerebra and cerebella of *Toxocara*-infected mice [29]. According to Janecek et al. [18], invasion of *Toxocara* larvae into the brain occurs in a biphasic pattern, at day 7 as well as day 42 pi. Therefore, the first migration wave may provoke a reactive anti-inflammatory response due to inflammatory processes caused by larval migration. The increased larval burden due to the second migration wave, and therefore an enhanced potential of parasite-host interactions and host manipulations by secreted E/S products, may be responsible for the decrease of pro- as well as anti-inflammatory cytokines in the chronic phase, relative to previous levels.

In addition to cytokines, chemokines and their corresponding receptors play plurifunctional roles in the brain. They are involved in phagocytosis, apoptosis and cytokine secretion during inflammatory processes, but are mainly known for their chemotactic activity and recruitment of effector cells such as monocytes, granulocytes and effector T cells to sites of inflammation [48]. Furthermore, neurons and astrocytes in cerebral tissues can release MCP-1 (CCL2) after pathogen stimulation or brain injury or trauma [49–51]. Noteworthy, levels of MCP-1 were not affected in the present study. In contrast, pro-inflammatory MIP-1α (CCL3) but not MIP-1β (CCL4) was consistently elevated in both brain parts of *T*. *canis*-infected as well as the cerebella of *T*. *cati*-infected mice. This chemokine is secreted by activated macrophages, acts as an attractant for polymorphic leukocytes and induces the secretion of pro-inflammatory cytokines [52]. The same pattern was detected for eotaxin (CCL11). Previous experiments suggest that eotaxin and IL-5 act cooperatively in regulating blood and tissue eosinophilia. IL-5, which peaked during the acute and subacute phase in the present study, is essential for the mobilisation of eosinophils from bone marrow, while eotaxin induces a rapid recruitment of eosinophils to inflamed tissue in a more localised fashion [53, 54]. Observed patterns of these two mediators (probably) support eosinophilic meningitis as a hallmark of NT [8, 9].

A single metabolite, the chemokine RANTES (CCL5), was exclusively affected in the cerebellum, where both infection groups showed elevated levels during the subacute and chronic phase of NT. The molecule and its receptor CCR5 have been associated with a variety of neuropathological conditions, including multiple sclerosis, stroke and Parkinson’s disease [46, 55, 56]; however, the definitive role of RANTES in the diseased CNS remains unclear [57].

## Conclusions

The results of this study provide new insights into the cerebral immune response and the induction of different cyto-/chemokines during *T*. *canis*- and *T*. *cati*-induced NT. Overall, only minor alterations of pro-inflammatory cytokines were detected in the acute und subacute phase of infection, followed by a decrease during the chronic phase. The chemokine MIP-1α was the only elevated pro-inflammatory mediator. Generally, it is important to keep in mind when viewing cytokine and chemokine data of the presented study that NT is characterised by focally distributed lesions in the CNS, but for technical reasons homogenates of the entire cerebra and cerebella were processed in the current analysis. Thus, healthy brain tissue was overrepresented compared to affected tissue, possibly downsizing or masking local effects. Nevertheless, the two *Toxocara* species often revealed different effects on the cyto- and chemokine pattern in brains of infected paratenic hosts, reflecting the described species-specific tropism to different brain parts in paratenic hosts. These results may indicate a parasite-induced immunomodulatory effect to evade the hosts’ immune response, facilitating persistence in the brain.

## Additional files

Additional file 1: Body and brain masses as well as brain-body mass ratios during the course of neurotoxocarosis. Additional file 1 shows body and brain masses as mean ± SD in [mg] as well as the brain-body mass ratios in uninfected control mice and *T. canis*- and *T. cati*-infected mice (*n* = 5). *P*-values of conducted t-test or Mann-Whitney U test between infected and uninfected animals are listed. (XLSX 104 kb)

Additional file 2: Concentrations of analysed cytokines and chemokines in cerebra of *T. canis*-, *T. cati*- and uninfected control mice. Additional file 2 shows the concentration of analysed cyto−/chemokines as mean ± SD in [pg/ml] in the cerebrum of uninfected control mice and *T. canis*- and *T. cati*-infected mice (n = 5). *P*-values of conducted t-test or Mann-Whitney U test between infected and uninfected animals are listed. (XLSX 20 kb)

Additional file 3: Concentrations of analysed cytokines and chemokines in cerebella of *T. canis*-, *T. cati*- and uninfected control mice. Additional file 3 shows the concentration of analysed cyto−/chemokines as mean ± SD in [pg/ml] in the cerebellum of uninfected control mice and *T. canis*- and *T. cati*-infected mice (n = 5). *P*-values of conducted t-test or Mann-Whitney U test between infected and uninfected animals are listed. (XLSX 20 kb)

## Data Availability

All data generated or analysed during this study are included in this published article.

## Abbreviations

CCL: C-C motif ligand
CNS: Central nervous system
CXCL: C-X-C motif ligand
G-CSF: Granulocyte-colony stimulating factor
GM-CSF: Granulocyte-macrophage colony-stimulating factor
IFN-γ: Interferon-gamma
IL: Interleukin
KC: Keratinocyte chemoattractant
L3: Third stage larvae
MCP-1: Monocyte chemoattractant protein 1
MIP-1: Macrophage inflammatory protein 1
NT: Neurotoxocarosis
pi: *Post infectionem*
RANTES: Regulated on activation, normal T cell expressed and secreted
*T*.: *Toxocara*
TES: *T*. *canis* excretory–secretory
TNF-α: Tumour necrosis factor-alpha
